# Supplementary material for: Combined MITOchondrial-NUCLEAR (MITO-NUCLEAR) Analysis for Mitochondrial Diseases Diagnosis: Validation and Implementation of a One-Step NGS Method
Source: Genes (Basel). 2023 May 15;14(5):1087. doi: 10.3390/genes14051087 (PMC10217848; doi:10.3390/genes14051087)
Supplement: Supplementary file 1 [file genes-14-01087-s001.zip › Legends at Supplementary files.pdf]

Table S1. The table shows all the nuclear and mitochondrial variants detected with MITO-NUCLEAR NGS assay. Furthermore, mtDNA variants, previously detected with Sanger method are shown, also. In bold are reported the mitochondrial variants detected with the NGS method but not with the Sanger sequencing. Molecular data for each patient (S1, S2, S3, S4, S5) and for the case report are available in the different sheets. In particular, for the S3 sample the table shows the overlapping molecular data from Clinical Exome CCP17 and the nuclear genes of the MITO-NUCLEAR. ACMG*:* American College of Medical Genetics and Genomics. The GB frequency data is derived from 59389 GenBank sequences with size greater than 15.4kbp and 78884 Control Region sequences with size 0.4-1.6kbp (http://www.mitomap.org/MITOMAP/GBFreqInfo).

Figure S1. The figure shows the NGS report of patient S2 and the graphical representation of the heteroplasmic variant with its percentage **(a)**. It's also shows the NGS report of patient S5 and the graphical representation of the heteroplasmic variant with the relative percentage **(b)**. *RD* (read depth).

Table S2. List of nuclear genes investigated by MITO-NUCLEAR NGS assay
