# Supplementary figures and images for: Combined MITOchondrial-NUCLEAR (MITO-NUCLEAR) Analysis for Mitochondrial Diseases Diagnosis: Validation and Implementation of a One-Step NGS Method
Source: Genes (Basel). 2023 May 15;14(5):1087. doi: 10.3390/genes14051087 (PMC10217848; doi:10.3390/genes14051087)

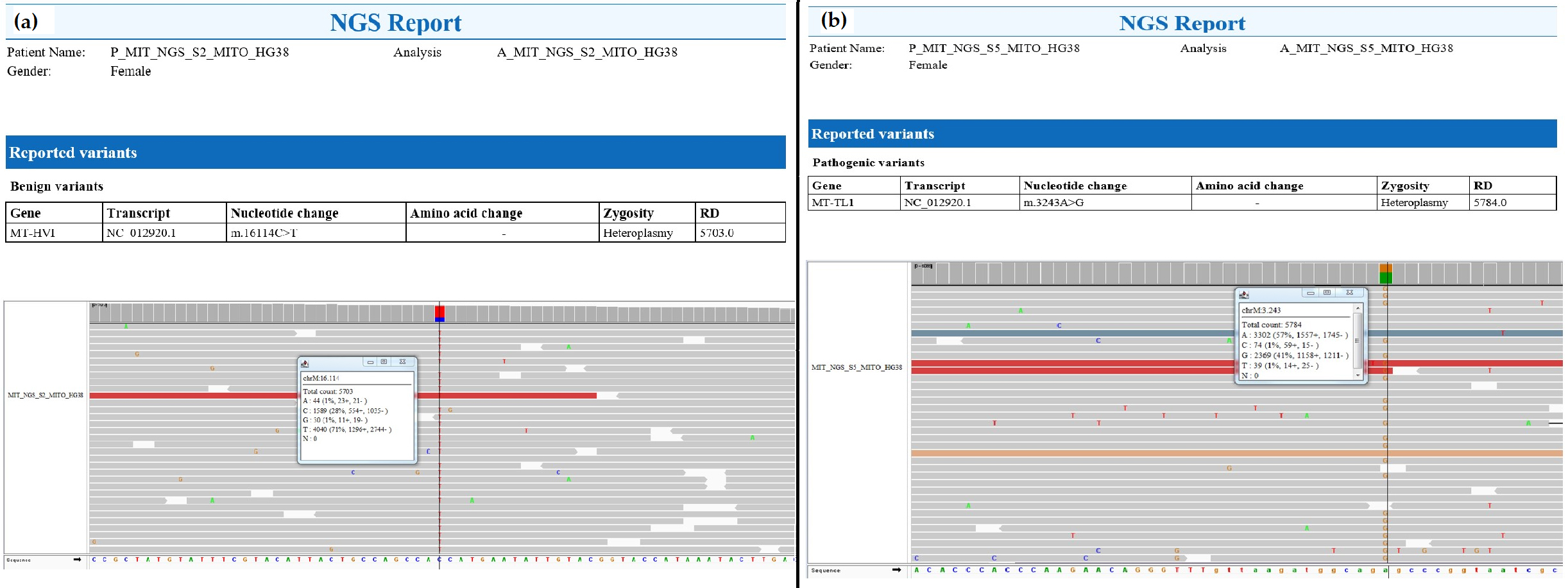

Supplement: Supplementary file 1 [file genes-14-01087-s001.zip › genes-2325390-supplementary-Figure S1.jpg]
